# Mean Apparent Propagator MRI Is Better Than Conventional Diffusion Tensor Imaging for the Evaluation of Parkinson’s Disease: A Prospective Pilot Study

**DOI:** 10.3389/fnagi.2020.563595

**Published:** 2020-09-24

**Authors:** Hongbo Le, Weike Zeng, Huihong Zhang, Jianing Li, Xiaoyan Wu, Mingwei Xie, Xu Yan, Minxiong Zhou, Huiting Zhang, Mengzhu Wang, Guobin Hong, Jun Shen

**Affiliations:** ^1^Department of Radiology, The Fifth Affiliated Hospital, Sun Yat-sen University, Zhuhai, China; ^2^Department of Radiology, Sun Yat-sen Memorial Hospital, Sun Yat-sen University, Guangzhou, China; ^3^Food Safety and Health Research Center, School of Public Health, Southern Medical University, Guangzhou, China; ^4^MR Scientific Marketing, Siemens Healthcare, Shanghai, China; ^5^College of Medical Imaging, Shanghai Key Laboratory of Molecular Imaging, Shanghai University of Medicine and Health Science, Shanghai, China

**Keywords:** Parkinson’s disease (PD), magnetic resonance imaging (MRI), mean apparent propagator (MAP), diffusion tensor imaging (DTI), gray matter

## Abstract

**Background and Purpose:**

Mean apparent propagator (MAP) MRI is a novel diffusion imaging method to map tissue microstructure. The purpose of this study was to evaluate the diagnostic value of the MAP MRI in Parkinson’s disease (PD) in comparison with conventional diffusion tensor imaging (DTI).

**Methods:**

23 PD patients and 22 age- and gender-matched healthy controls were included. MAP MRI and DTI were performed on a 3T MR scanner with a 20-channel head coil. The MAP metrics including mean square displacement (MSD), return to the origin probability (RTOP), return to the axis probability (RTAP), and return to the plane probability (RTPP), and DTI metrics including fractional anisotropy (FA), and mean diffusivity (MD), were measured in subcortical gray matter and compared between the two groups. The receiver operating characteristic (ROC) curve was used to analyze the diagnostic performance of all the metrics. The association between the diffusion metrics and disease severity was assessed by Pearson correlation analysis.

**Results:**

For MAP MRI, the mean values of MSD in the bilateral caudate, pallidum, putamen, thalamus and substantia nigra (SN) were higher in PD patients than in healthy controls (*p*_FDR_ ≤ 0.001); the mean values of the zero displacement probabilities (RTOP, RTAP, and RTPP) in the bilateral caudate, pallidum, putamen and thalamus were lower in PD patients (*p*_FDR_ < 0.001). For DTI, only FA in the bilateral SN was significantly higher in PD patients than those in the controls (*p*_FDR_ < 0.001). ROC analysis showed that the areas under the curves of MAP MRI metrics (MSD, RTOP, RTAP, and RTPP) in the bilateral caudate, pallidum, putamen and thalamus (range, 0.85–0.94) were greater than those of FA and MD of DTI (range, 0.55–0.69) in discriminating between PD patients and healthy controls. RTAP in the ipsilateral pallidum (*r* = −0.56, *p*_FDR_ = 0.027), RTOP in the bilateral and contralateral putamen (*r* = −0.58, *p*_FDR_ = 0.019; *r* = −0.57, *p*_FDR_ = 0.024) were negatively correlated with UPDRS III motor scores.

**Conclusion:**

MAP MRI outperformed the conventional DTI in the diagnosis of PD and evaluation of the disease severity.

## Introduction

Parkinson’s disease (PD) is the second most common neurodegenerative disorder affecting 2–3% of the population ≥65 years of age (Poewe et al., 2017). Degeneration of nigrostriatal dopaminergic neurons results in disruption of basal ganglia–thalamocortical loops, which underlies the classical motor signs and symptoms of PD (Armstrong and Okun, 2020). So far, PET and single-photon emission CT (SPECT) remain the mainstay imaging approaches for the early and differential diagnosis of PD (Poewe et al., 2017; Armstrong and Okun, 2020). Whereas, SPECT and PET require radioactive tracers and the limited availabilities impede its widespread application. Alternatively, diffusion tensor imaging (DTI) has been used to detect and quantify neurodegeneration in PD (Cochrane and Ebmeier, 2013; Hall et al., 2016; Atkinson-Clement et al., 2017). Decreased fractional anisotropy (FA) and/or increased mean diffusivity (MD) in gray and white matter regions, particularly in subcortical areas such as the substantia nigra (SN), the putamen, the pallidum, and the caudate, have been reported (Atkinson-Clement et al., 2017).

Although DTI appears to be a sensitive method to study PD, it has an inherent strong modeling constraint, that is, this method presumes that water diffusion in the brain is not restricted and follows Gaussian distribution (Lee et al., 2018). However, water molecular diffusion often follows a non-Gaussian distribution in the complex biological tissues, due to the restriction of cell membranes, organelles, and liquid compartments (Khairnar et al., 2017). These microstructures cannot be reflected by DTI models and may also introduce bias in its metrics. A recently developed diffusion model, called mean apparent propagator (MAP) MRI, can overcome this limitation. The MAP MRI model does not make any prior assumptions about the behavior of the water diffusion in the tissues (Hosseinbor et al., 2013; Olson et al., 2019). This method models broader *q*-space signals utilizing Hermite polynomials (Özarslan et al., 2013). As a result, more subtle changes in complex microstructures could be investigated by the MAP MRI metrics than the DTI (Özarslan et al., 2013; Olson et al., 2019). For MAP MRI, the mean square displacement (MSD) can be derived from the diffusion propagator models to measure the average amount of diffusion, and zero displacement probabilities, including the return to the origin probability (RTOP), the return to the axis probability (RTAP) and the return to the plane probability (RTPP), can be derived to quantify various features of the three-dimensional diffusion process (Hosseinbor et al., 2013; Özarslan et al., 2013; Ning et al., 2015; Fick et al., 2016). RTOP estimated from three-dimensional *q*-space data is a metric that indicates the likelihood of water molecules undergoing zero net displacements between the applications of the two diffusion sensitizing gradients (Özarslan et al., 2013; Zucchelli et al., 2016). If the zero net displacement probabilities of diffusion molecules are high in a region, the RTOP value is high and it appears hyperintense. This hyperintensity is mainly in white matter because the white matter has more restricting barriers (e.g., multilayer myelin sheaths, axonal membranes, and microtubules). If the zero net displacement probabilities are low in a region, the RTOP is low and it appears hypointense. The hypointensity is mainly in cerebrospinal fluid because it is free water. The RTAP and RTPP are the variants of RTOP in two-dimensions and one-dimension, respectively. RTAP is decomposed from RTOP along the perpendicular to the direction of the primary eigenvector, and reflect the presence of restrictive barriers in the radial orientation; RTPP is decomposed along the parallel and reflects the presence of restrictive barriers in the axial orientation (Özarslan et al., 2013; Zucchelli et al., 2016).

Recently, MAP MRI indices have been proved the clinical availability in several neuroradiological studies (Avram et al., 2016; Boscolo Galazzo et al., 2018; Ma et al., 2020). Avram et al. (2016) demonstrated that MAP MRI microstructural parameters have significant neuroanatomical consistency across healthy controls and reproducibility in test-retest experiments. The RTOP, RTAP, RTPP, and MSD achieved a high predictive power for clinical outcome over cortico-subcortical connections and good discrimination between stroke patients and controls at different time scales (Boscolo Galazzo et al., 2018). Ma et al. (2020) reported that the RTPP had the strongest effect size for differentiating hippocampus ipsilateral to the epileptogenic focus from the contralateral hippocampus when compared to all other DTI/MAP MRI parameters, signal intensity on fluid attenuated inversion recovery (FLAIR) imaging and hippocampal volumes. They also found that the RTAP, RTOP, and MSD were negatively or positively correlated to clinical measures of delayed recall (Ma et al., 2020). However, there is a lack of studies to explore the use of MAP MRI in PD patients.

In this study, MAP metrics of subcortical gray matter including caudate, pallidum, putamen, thalamus, and SN, were measured, for the first time, in PD patients and the healthy controls in comparison with conventional DTI. The purpose of our study was to determine the role of MAP MRI in the diagnosis of PD and in the evaluation of disease severity.

## Materials and Methods

### Participants

**FIGURE 1:**
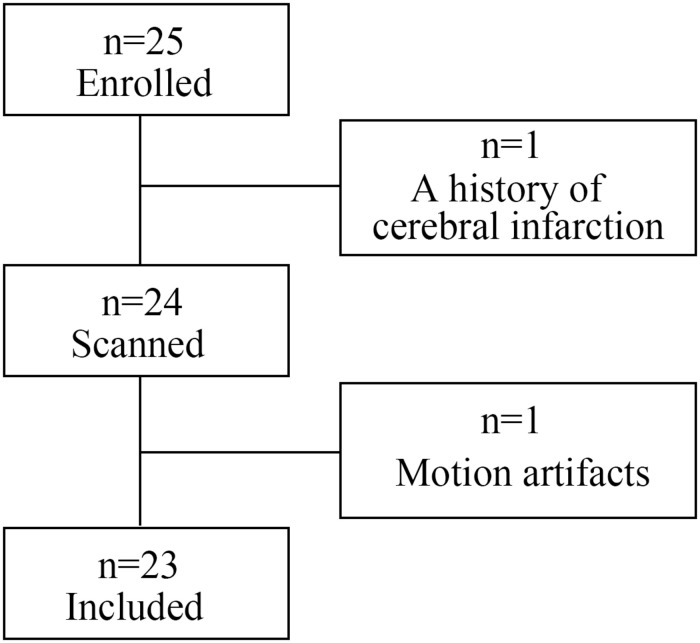
Patient flow diagram.

This prospective study was approved by the institutional research ethics board of Sun Yat-sen Memorial Hospital, Sun Yat-sen University (Guangzhou, China), and written informed consent was obtained from all participants. From January 2019 to January 2020, 25 consecutive patients with PD were enrolled. Patients were excluded from this study if they have other neuropsychiatric diseases including atypical parkinsonian disorders, contraindications to MRI scans, history of alcohol abuse, or extensive cranial organic disease, or if the image quality of MRI is ineligible for data analysis. One patient was excluded because of a history of cerebral infarction; another was excluded from later analysis due to excessive motion artifacts. Finally, 23 patients (15 males and 8 females; mean age 65.65 ± 8.39 years) were included in our study. The patient flow diagram is presented in Figure 1. All PD patients met the diagnostic criteria proposed by the Movement Disorder Society (Postuma et al., 2015) and atypical parkinsonian disorders were excluded according to clinical feature, MRI and PET examinations based on internationally established operational criteria (Bak and Hodges, 2008; Gilman et al., 2008; Postuma et al., 2015; Höglinger et al., 2017). The demographic information of the study participants is shown in Table 1. Disease duration was defined as the number of years between the first reported motor symptoms and the study visit date. Hoehn and Yahr staging scale (H&Y), Unified Parkinson’s Disease Rating Scale part III (UPDRS-III) and Montreal Cognitive Assessment (MoCA) examinations were performed to assess disease stage, motor function and cognitive status. Eight patients were treated by monotherapy with Levodopa-Benserazide; 11 patients were treated with Levodopa-Benserazide and Pramipexole; 4 patients were treated with Levodopa-Benserazide, Pramipexole, and Entacapone. Levodopa equivalent daily dose (LEDD) was calculated for PD patients (Tomlinson et al., 2010). All patients were on antiparkinson medication at the time of testing. UPDRS-III, H&Y and MoCA examinations were performed during “on” medication state (approximately 1 h after the last dose of medication) the day before MRI scan. On the day of MRI scanning, patients took their medications as usual, and 1 h later MRI was performed when they were fully responding to their PD medications. Twenty-two age- and gender-matched healthy controls with no history of neuropsychiatric diseases were also included.

**TABLE 1:** Demographics for of PD patients and healthy controls.

	Control group (*n* = 22)	PD group (*n* = 23)	*P*-value
Sex, male/female	14/8	15/8	0.912
Age, years	63.64 ± 6.89	65.65 ± 8.39	0.384
Disease duration, years	NA	5.49 ± 3.86	NA
UPDRS-III motor score	NA	21.74 ± 6.74	NA
H&Y stage	NA	2.11 ± 0.66	NA
MoCA	NA	24.57 ± 2.51	NA
LEDD	NA	559.40 ± 263.70	NA

*Age, disease duration, UPDRS-III motor score and H&Y stage represent by mean ± SD. P-value is based on the Chi-square test for gender and the independent samples t-test for age. MoCA, montreal cognitive assessment; LEDD: levodopa equivalent daily dose; NA, not applicable.*

### MR Imaging Protocol

All participants underwent brain MRI examination. MRI was performed on a 3T MR scanner (MAGNETOM Skyra; Siemens Healthcare, Erlangen, Germany) with a 20-channel head coil. A standardized protocol, including T2-weighted imaging (T2WI), T1-weighted imaging (T1WI), FLAIR, diffusion-weighted imaging (DWI), and 3D-T1WI, was used for all participants. T2WI, T1WI, and FLAIR were acquired to rule out other neurologic disorders such as moderate to severe white matter disease, stroke and brain tumor. The detailed acquisition parameters are shown in Table 2. DWI data were acquired by using a half coverage Cartesian *q*-space grid scheme with a radial grid size of 4. Sixteen *b*-values (*b* = 100, 150, 250, 400, 500, 650, 750, 1000, 1100, 1150, 1250, 1400, 1500, 1650, 1750, and 2000 s/mm^2^) along 128 diffusion gradient directions were included in the acquisition, and one image was acquired at *b* = 0 s/mm^2^. The small/large diffusion time was 33.9/43.4 ms.

**TABLE 2:** Imaging sequences and acquisition parameters of MRI.

	Sequence	TR/TE/TI (ms)	Slice thickness/gap (mm)	FOV (mm^2^)	Voxel size (mm^3^)	Acquisitio*n* time (min)
T2WI	TSE	4500/96/-	5/1	220 × 220	0.25 × 0.25 × 6	1:39
T1WI	TSE	2000/9/900	5/1	220 × 220	0.69 × 0.69 × 6	1:22
FLAIR	TSE	9000/99/2500	5/1	220 × 220	0.69 × 0.69 × 6	2:08
DWI	SE-EPI	4900/93/-	4/0.4	220 × 220	2 × 2 × 4.4	12:17
3D-T1WI	MPRAGE	1900/2.3/900	0.9/0	230 × 230	0.9 × 0.9 × 0.9	5:11

*T2WI, T2-weighted images; T1WI, T1-weighted images; FLAIR, fluid-attenuated inversion recovery; DWI, diffusion weighted images; TSE, turbo spin-echo; SE-EPI, spin-echo echo-planar imaging; MPRAGE, magnetization-prepared rapid acquisition gradient-echo; TR, repetition time; TE, echo time; TI, inversion time; FOV, field of view.*

### Image Processing and ROI Segmentation

All DWI data underwent eddy current and motion correction via the Diffusionkit tool (Xie et al., 2016). Then, MAP MRI parameter fitting was performed by using a software tool, NeuDiLab, which was developed in-house based on an open-resource tool DIPY (Diffusion Imaging in Python,^1^) (Garyfallidis et al., 2014). MAP MRI parameters included MSD, RTOP, RTAP, and RTPP, and DTI parameters included FA and MD. Afterward, all the 3D T1WI, MAP MRI, DTI, and b0 maps were post-processed by using SPM12^2^. The steps were as follows. Firstly, the 3D T1WI maps were co-registered to the standard space, as defined by the Montreal Neurological Institute (MNI) T1-weighted template. Secondly, the individual b0, MAP MRI, and DTI maps were co-registered to the respective 3D T1WI structural imaging. Finally, all the parameter maps were spatially normalized to MNI space by using the non-linear registration, with a voxel size of 2 mm × 2 mm × 2 mm.

**FIGURE 2:**
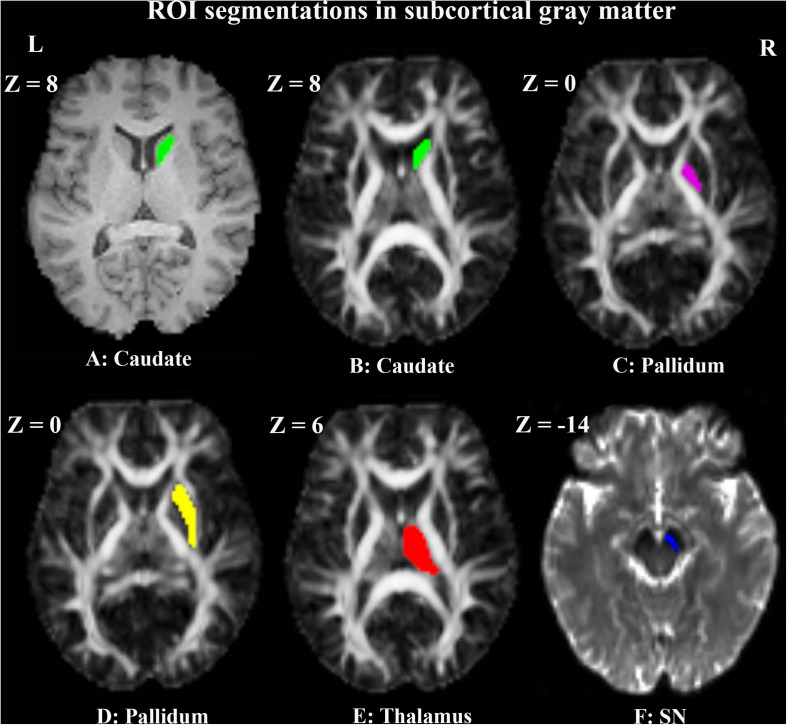
Example of manual ROI segmentations in the right subcortical gray matter of a representative PD patient. The ROI of the right caudate is shown in green on T1WI **(A)** and FA map **(B)**. The ROIs of right pallidum **(C)**, putamen **(D)**, and thalamus **(E)** are shown in violet, yellow and red on FA maps, respectively. The ROI of right SN **(F)** is shown in blue on the b0 map.

Two neuroradiologists, who were blinded to the clinical data, conducted the manual region of interest (ROI) segmentations independently according to the MRI brain atlas (Cho, 2015) by the use of MRIcron^3^. The ROIs of caudate, pallidum, putamen and thalamus (Lee et al., 2011) were drawn on normalized T1WI, while toggling among the FA and b0 maps to avoid contamination by adjacent white matter and cerebrospinal fluid. The ROIs of SN were drawn on normalized b0 maps, while checking in parallel the overlay on the T1WI and FA maps to ensure the exclusion of non-gray matter voxels. The ROIs were drawn on the slices where the structures were most prominent. The right ROI of each nucleus was firstly segmented. Then the left ROI was segmented in the mirror region at the same axial slice with the same number of voxels. Contiguous 4 slices (*Z* = 4, 6, 8, 10), 3 slices (*Z* = −2, 0, 2), 5 slices (*Z* = −2, 0, 2, 4, 6), 5 slices (*Z* = 4, 6, 8, 10, 12) and 3 slices (*Z* = −10, −12, −14) were chosen for the ROIs of caudate, pallidum, putamen, thalamus and SN, respectively. The ROI voxels for a unilateral caudate, pallidum, putamen, thalamus and SN were 70, 60, 220, 220, and 25, respectively. The positions of the ROIs in a representative PD patient are shown in Figure 2.

### Statistical Analysis

Age, disease duration, UPDRS-III motor score, H&Y stage and parameter values are represented as mean values ± SD, unless otherwise noted. The intraclass correlation coefficient (ICC) was calculated to assess the interrater reliability between two raters in the manual ROI segmentation. ICC results of all the measured parameters showed good agreement between the two neuroradiologists (ICC ≥ 0.917, *p* < 0.001). The mean values of the two measurements in the ROIs were used in the statistical analysis. The independent samples *t*-test was used to compare the age and diffusion parameter values between the two groups. The Chi-square test was used to compare gender differences. The receiver operating characteristic (ROC) curve and the areas under the ROC curve (AUCs) were used to compare the overall diagnostic performance of the diffusion parameters in each nucleus. The associations between the diffusion parameters and clinical status of the disease were assessed by using Pearson correlation coefficients. All statistical analyses were performed by using SPSS version 22.0 (IBM Corp., Armonk, NY, United States). Two-tailed *P* < 0.05 was considered to be statistically significant. The Benjamini-Hochberg correction was used to control the false discovery rate (FDR) for multiple hypothesis testing (Benjamini and Hochberg, 1995). FDR-corrected *P* < 0.05 was considered significant and uncorrected *P* < 0.05 was considered as trend.

## Results

### MAP MRI and DTI Maps

**FIGURE 3:**
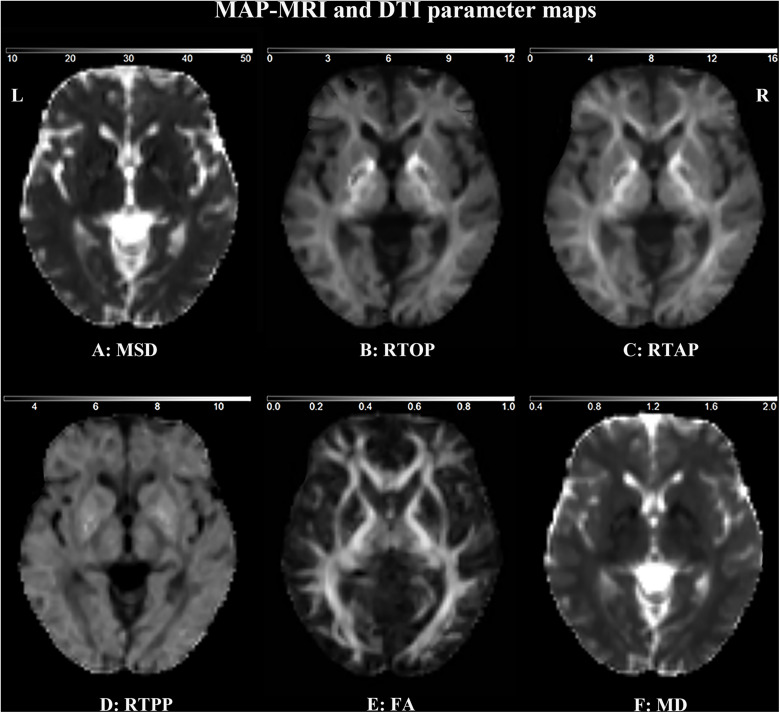
MAP MRI and DTI parameter maps of a representative healthy subject. MSD **(A)**, RTOP **(B)**, RTAP **(C)** and RTPP **(D)** are derived from MAP MRI, and FA **(E)**, and MD **(F)** are derived from DTI. FA is dimensionless; MD, MSD, RTOP, RTAP, and RTPP are expressed in 10^–3^ mm^2^ s^–1^, 10^–5^ mm^2^, 10^5^ mm^–3^, 10^3^ mm^–2^, and 10^1^ mm^–1^, respectively.

MAP MRI and DTI maps in a representative healthy control are shown in Figure 3. The white matter appears hypointense and the free water (e.g., cerebrospinal fluid) appears hyperintense on the MSD and MD maps (Figures 3A,F). On the contrary, the white matter appears hyperintense and the free water appears hypointense on the RTOP, RTAP, and RTPP maps (Figures 3B–E).

### MAP and DTI Metrics

**FIGURE 4:**
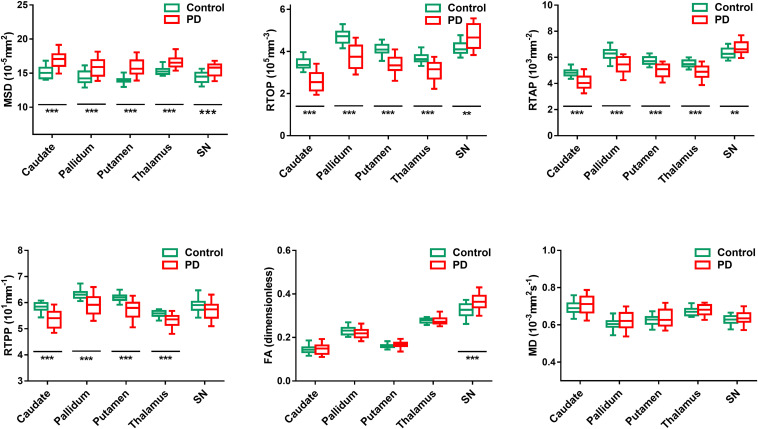
Comparison of MAP MRI (MSD, RTOP, RTAP, and RTPP) and DTI (FA and MD) values between PD patients (red) and healthy controls (green) in the bilateral subcortical gray matter. Significant differences between PD patients and controls are represented as: *p_*FDR*_ < 0.05, **p_*FDR*_ < 0.01, ***p_*FDR*_ < 0.001.

The diffusion parameter values of the bilateral caudate, pallidum, putamen, thalamus, and SN in each group are shown in Figure 4. FA is dimensionless; MD, MSD, RTOP, RTAP, and RTPP are expressed in 10^–3^ mm^2^ s^–1^, 10^–5^ mm^2^, 10^5^ mm^–3^, 10^3^ mm^–2^, and 10^1^ mm^–1^, respectively (Fick et al., 2016). For MAP MRI parameters, the mean MSD of all measured subcortical gray matter was significantly higher in PD patients than in healthy controls (*p*_FDR_ < 0.001). The mean values of the zero displacement probabilities (RTOP, RTAP, and RTPP) in the bilateral caudate, pallidum, putamen and thalamus were significantly lower in PD patients (*p*_FDR_ < 0.001), while the mean values of RTOP and RTAP of the bilateral SN were higher (*p*_FDR_ < 0.003) in PD patients than in healthy controls. For DTI parameters, only the mean value of FA in the bilateral SN was significantly higher in PD patients than in controls (*p*_FDR_ < 0.001). There were no significant differences in other parameter values in the measured ROIs between the two groups.

### Diagnostic Performance

**FIGURE 5:**
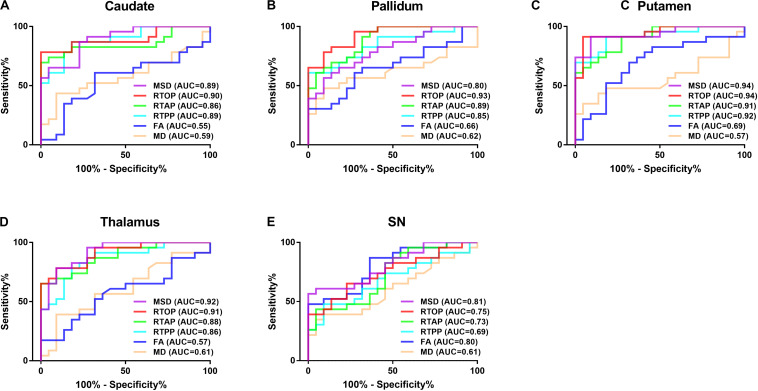
The Receiver operating characteristic (ROC) analysis of MAP MRI and DTI parameters in the bilateral caudate **(A)**, pallidum **(B)**, putamen **(C)**, thalamus **(D)** and SN **(E)**. MAP MRI showed higher diagnostic performances than DTI in distinguishing PD patients from controls. AUC: the area under the curve.

The results of the ROC curve analysis are presented in Figure 5 and Table 3. The AUCs of MAP MRI metrics including MSD, RTOP, RTAP, and RTPP in the bilateral caudate, pallidum, putamen and thalamus (range, 0.85–0.94) were greater than the counterparts of FA and MD of DTI (range, 0.55–0.69), which indicated that the former had better performance than the latter in distinguishing PD patients from healthy controls. The best diagnostic performance was achieved by RTOP of the contralateral side of the putamen (AUC = 0.95). The AUCs of all measured MAP MRI and DTI metrics in the SN were ranged from 0.60 to 0.82.

**TABLE 3:** Areas under ROC Curve for parameter values.

ROI	MSD	RTOP	RTAP	RTPP	FA	MD
Caudate						
Contralateral	0.91	0.90	0.87	0.87	0.56	0.59
Ipsilateral	0.89	0.94	0.84	0.88	0.60	0.58
Bilateral	0.89	0.90	0.86	0.89	0.55	0.59
Pallidum						
Contralateral	0.78	0.94	0.87	0.85	0.66	0.64
Ipsilateral	0.80	0.91	0.87	0.82	0.61	0.60
Bilateral	0.80	0.93	0.89	0.85	0.66	0.62
Putamen						
Contralateral	0.94	0.95	0.89	0.89	0.70	0.58
Ipsilateral	0.93	0.91	0.89	0.91	0.74	0.55
Bilateral	0.94	0.94	0.91	0.92	0.69	0.57
Thalamus						
Contralateral	0.91	0.90	0.88	0.83	0.56	0.62
Ipsilateral	0.91	0.90	0.87	0.81	0.53	0.58
Bilateral	0.92	0.91	0.88	0.86	0.57	0.61
SN						
Contralateral	0.79	0.78	0.72	0.68	0.80	0.62
Ipsilateral	0.80	0.73	0.74	0.62	0.82	0.60
Bilateral	0.81	0.75	0.73	0.69	0.80	0.61

### Correlations Between Diffusion Parameters Values and Disease Severity

**FIGURE 6:**
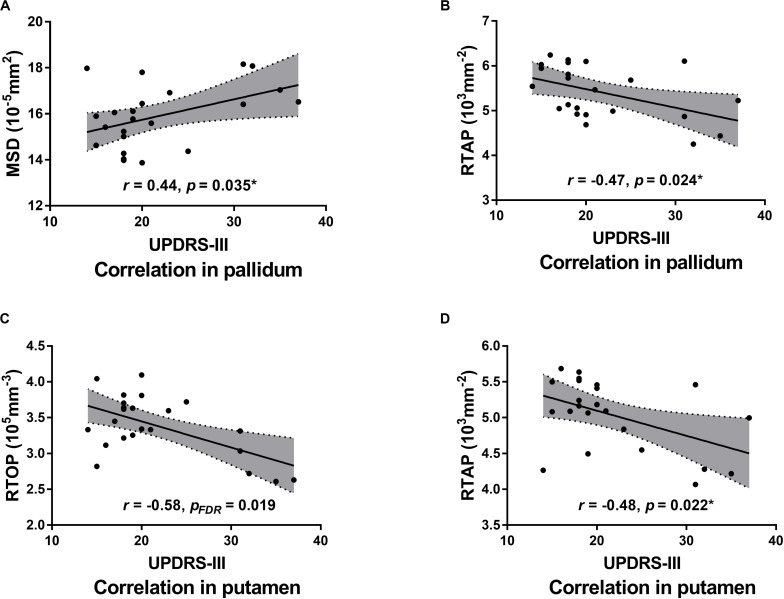
Scatter plots of the significant correlations between MAP MRI measurements and UPDRS-III motor scores (“on” medication state) in the bilateral pallidum **(A,B)** and putamen **(C,D)**. Pearson correlation coefficients and associated *p*-values are shown. The shaded areas represent the 95% confidence interval for each regression line. *Uncorrected.

Pearson correlation analysis showed that MSD of the bilateral and ipsilateral pallidum (*r* = 0.44, uncorrected *p* = 0.035; *r* = 0.47, uncorrected *p* = 0.023) were positively correlated with UPDRS III motor scores, but none of the results survived FDR correction; RTAP of the bilateral and ipsilateral pallidum (*r* = −0.47, uncorrected *p* = 0.024; *r* = −0.56, *p*_FDR_ = 0.027), RTAP of the bilateral and contralateral putamen (*r* = −0.48, uncorrected *p* = 0.022; *r* = −0.48, uncorrected *p* = 0.020), and RTOP of the bilateral, contralateral and ipsilateral putamen (*r* = −0.58, *p*_FDR_ = 0.019; *r* = −0.57, *p*_FDR_ = 0.024; *r* = −0.51, uncorrected *p* = 0.014) were negatively correlated with UPDRS III motor scores. Results of correlation analyses between parameter values and UPDRS-III scores are presented in Table 4 and Figure 6. No statistically significant correlation was found between other parameters and the clinical status of PD including UPDRS-III scores, H&Y stage, disease duration, and MoCA scores.

**TABLE 4:** Pearson correlation analysis between parameters values and UPDRS III scores.

	MSD		RTOP		RTAP		RTPP		FA		MD	
ROI	R	*P*_FDR_	r	*P*_FDR_	r	*P*_FDR_	r	*P*_FDR_	r	*P*_FDR_	r	*P*_FDR_
Caudate												
Contralateral	0.103	0.694	−0.032	0.886	−0.339	0.284	−0.222	0.708	−0.259	0.583	0.199	0.362
Ipsilateral	0.011	0.959	−0.067	0.760	−0.288	0.304	−0.234	0.515	−0.184	0.761	0.218	0.440
Bilateral	0.055	0.804	−0.049	0.825	−0.315	0.239	−0.240	0.450	−0.225	0.709	0.211	0.390
Pallidum												
Contralateral	0.357	0.199	−0.314	0.181	−0.319	0.229	−0.445	0.167	−0.098	0.819	0.340	0.281
Ipsilateral	0.472	0.023**^†^**	−0.156	0.598	−0.562	0.027*	−0.222	0.515	−0.063	0.774	0.292	0.441
Bilateral	0.441	0.035**^†^**	−0.231	0.360	−0.469	0.024**^†^**	−0.347	0.450	−0.082	0.709	0.318	0.349
Putamen												
Contralateral	0.334	0.199	−0.566	0.024*	−0.481	0.020^†^	−0.139	0.659	−0.079	0.819	0.205	0.362
Ipsilateral	0.206	0.649	−0.506	0.014**^†^**	−0.405	0.138	−0.171	0.543	−0.126	0.761	0.169	0.440
Bilateral	0.271	0.351	−0.580	0.019*	−0.475	0.022**^†^**	−0.165	0.566	−0.106	0.709	0.188	0.390
Thalamus												
Contralateral	0.360	0.199	−0.418	0.118	−0.044	0.857	0.083	0.708	−0.050	0.819	0.356	0.281
Ipsilateral	0.142	0.649	−0.321	0.253	−0.154	0.604	0.343	0.515	−0.113	0.761	0.302	0.441
Bilateral	0.258	0.390	−0.382	0.181	−0.103	0.799	0.243	0.450	−0.083	0.709	0.332	0.349
SN												
Contralateral	0.087	0.694	0.354	0.162	−0.040	0.857	−0.156	0.708	−0.270	0.583	−0.215	0.362
Ipsilateral	0.176	0.649	0.309	0.253	0.012	0.958	−0.033	0.883	−0.270	0.761	−0.195	0.440
Bilateral	0.133	0.684	0.335	0.198	−0.016	0.943	−0.098	0.657	−0.272	0.709	−0.207	0.390

*Significant differences are represented as: ^∗^p_FDR_ < 0.05; ^†^Uncorrected.*

## Discussion

MAP MRI metrics of subcortical gray matter were measured in this study, for the first time, in PD patients and compared with conventional DTI. Our study showed that the MAP MRI metrics in most measured subcortical gray matter structures in PD patients were significantly different from those in the controls. The MAP MRI parameters of the deep gray matter except for SN had better diagnostic performance than DTI. MSD and RTAP values in the pallidum, as well as RTOP and RTAP in the putamen, were correlated with UPDRS III motor scores.

MAP MRI is a novel diffusion model based on *q*-space sampling and Hermite function computation (Özarslan et al., 2013). A previous study reported that the family of zero displacement probabilities including RTOP, RTAP, and RTPP, might reflect tissue microstructure changes better than the metrics derived from DTI (Avram et al., 2016). Our results also demonstrated that the zero displacement probabilities are more accurate and robust in assessing pathological microstructure than DTI parameters in PD. Furthermore, the combined exploitation of RTAP and RTPP could indicate specific pathological changes. If both RTAP and RTPP diminish, it indicates neuronal density reduction; if RTAP decreases, but RTPP increases, it indicates crossing fibers. As aforementioned, the zero displacement probabilities can indicate whether the metric changes are caused by crossing fibers or by a neural decrease (Zucchelli et al., 2016). In our study, RTAP and RTPP all decreased in the basal ganglia in PD patients compared with healthy controls. This may be associated with the degeneration of the projection neurofibers/axons of nigrostriatal dopaminergic neurons that are progressively lost in PD patients.

There is another explanation for RTAP changes. Previous studies have reported that RTAP is inversely related to the apparent diameter of the axon (Ong et al., 2008; Özarslan et al., 2013; Fick et al., 2016). The pathological hallmark of PD is axonal damage in the form of α-synuclein aggregates, axonal swellings, and dystrophic axonal profiles (Lundblad et al., 2012). Therefore, the reduction in RTAP may also due to an increase in axonal diameter as a result of axonal swelling.

MSD is another important MAP MRI metric. It represents the MSD of the water molecules in the unit time in a voxel and is proportional to the average amount of diffusion (Wu et al., 2008; Ning et al., 2015). MSD is closely related to the classical MD metric with sharing similar patterns (Wu and Alexander, 2007), but MSD captures higher-order statistics of the diffusion propagator and the *q*-space signal (Ning et al., 2015). Therefore, MSD is more sensitive to the hindered and restricted diffusion components than MD (Ning et al., 2015). In a previous stroke study, MSD was demonstrated to be more contrasted than MD inside the ischemic lesion, and MSD seems to identify and characterize different portions of the ischemic lesion, where MD appears to be more homogeneous (Boscolo Galazzo et al., 2018). In our study, the difference was observed in MSD but not in MD between PD patients and healthy controls, which indicates that MSD is superior to MD in investigating subtle abnormalities in the brain tissue of PD patients.

Metrics changes in SN are controversial. Previous studies have demonstrated conflicting observations in SN, such as an increased (Van Camp et al., 2009; Wang et al., 2011; Lenfeldt et al., 2015), decreased (Boska et al., 2007; Vaillancourt et al., 2009; Langley et al., 2016; Loane et al., 2016), or unaltered (Schuff et al., 2015; Hirata et al., 2017; Pelizzari et al., 2019) FA in PD patients. Our study showed an increased FA in SN in PD patients, which was consistent with some of the previous studies (Van Camp et al., 2009; Wang et al., 2011; Lenfeldt et al., 2015), but inconsistent with other studies (Boska et al., 2007; Vaillancourt et al., 2009; Langley et al., 2016; Loane et al., 2016). Notably, the increased FA in SN was consistent with the increased RTOP and RTAP in our study. In addition, the values of RTOP and RTAP in the SN were greater in PD patients than those in the controls, while these parameters of other subcortical gray matter areas were smaller in the PD patients. There are several explanations for this discrepancy. Firstly, levodopa has intrinsic magnetic properties that could induce artifactual signals (Atkinson-Clement et al., 2017). Secondly, SN ROI delineation may also affect DWI results in PD (Langley et al., 2015, 2016). Thirdly, the iron deposition in SN may disturb the intrinsic DWI signal, which may also lead to different results.

Although PD is a progressive neurodegenerative disorder characterized primarily by motor symptoms, PD patients can experience cognitive dysfunction at all stages of the disease (Schneider et al., 2015). In our study, the patients of our study were at mild to moderate PD stage, and MoCA scores indicated that they may be complicated with mild cognitive impairment (Dalrymple-Alford et al., 2010). In our cohort of mild to moderate PD, widespread changes were observed in subcortical gray matter including caudate, pallidum, putamen, thalamus and SN via MAP MRI metrics, while only DTI-derived FA detected abnormal changes in SN. Thus, MAP MRI may provide more sensitive biomarkers for detecting brain microstructural changes of PD compared to conventional DTI metrics.

Recently, a diffusion MRI analysis technique using a bi-tensor model was introduced, which allows the estimation of the fractional volume of free water within a voxel. By using this technique, free water in the posterior SN was found to elevate in PD patients compared to controls, and could persist over 1 year in PD patients (Ofori et al., 2015a, b, 2017; Planetta et al., 2016; Burciu et al., 2017). But this technique could not detect significant free-water or altered free-water corrected FA changes in the basal ganglia and thalamus (Planetta et al., 2016).

In our study, MAP MRI metrics of subcortical gray matter with greater AUCs performed better than the metrics of DTI except for RTOP, RTAP, and RTPP of SN; MSD and RTAP in the pallidum, together with RTOP and RTAP in the putamen were associated with motor functions. These results suggest that the MAP MRI parameters may be used as imaging biomarkers to distinguish PD patients from healthy controls and indicate disease severity for PD. Although PD patients generally have a unilateral disease onset, our results showed that the diagnostic performances of all the diffusion metrics from the contralateral, ipsilateral, and bilateral sides of subcortical gray matter were similar. This result may be attributed to a relatively long disease duration and mild to moderate stage of the PD in our study cohort.

There are several limitations to our study. First, the sample size of the prospective study is relatively small and the interpretability of the results is limited. In our study, although MAP MRI detected significant changes in the subcortical gray matter in PD patients, further multi-center study with a larger cohort are needed to show the utility of MAP-derived metrics. Second, ROI of SN was not separated into sub-regions (pars compacta and pars reticulata), which may decrease the ability of MAP MRI to assess PD. Third, UPDRS-III assessment was performed during “on” medication state and there may be a bias between the scores and pathological changes. Fourth, our study did not include longitudinal data and other parkinsonism (e.g., multiple system atrophy and progressive supranuclear palsy) data. Future studies will include these data to investigate whether MAP MRI could help monitor patients’ responses to neuroprotective or therapeutic clinical trials and differentiate PD from other parkinsonism.

## Conclusion

In conclusion, our preliminary study demonstrated that MAP MRI is better than conventional DTI for the diagnosis of PD and evaluation of the disease severity. MAP MRI parameters might be promising biomarkers for the diagnosis of PD and indicating disease severity.

## Data Availability Statement

The raw data supporting the conclusions of this article will be made available by the authors, without undue reservation.

## Ethics Statement

The studies involving human participants were reviewed and approved by the Ethics Committee of Sun Yat-sen Memorial Hospital, Sun Yat-sen University. The patients/participants provided their written informed consent to participate in this study.

## Author Contributions

HL, WZ, MW, GH, and JS conceived and designed the experiments. WZ, JL, and MX performed the experiments. HL, HHZ, XW, XY, MZ, HTZ, and MW analyzed the data and interpreted the results. HL, WZ, GH, and JS wrote and/or revised the manuscript. All authors contributed to the article and approved the submitted version.

## Conflict of Interest

XY, HZ, and MW were employed by the company MR Scientific Marketing, Siemens Healthcare. The remaining authors declare that the research was conducted in the absence of any commercial or financial relationships that could be construed as a potential conflict of interest.

## Footnotes

1 http://nipy.org/dipy

2 http://www.fil.ion.ucl.ac.uk/spm

3 https://www.nitrc.org/projects/mricron
